# Morphological and functional evaluation of the left ventricle in severe aortic stenosis with afterload mismatch: a South African single-centre, cross-sectional cardiovascular MRI-based study

**DOI:** 10.1136/bmjopen-2026-118642

**Published:** 2026-06-24

**Authors:** Megan R Rajah, Anton F Doubell, Philip G Herbst

**Affiliations:** 1 Division of Cardiology, Department of Medicine, Faculty of Medicine and Health Sciences, Tygerberg Hospital, Stellenbosch University, Cape Town, South Africa

**Keywords:** Valvular heart disease, Cardiovascular Disease, Magnetic resonance imaging, Adult cardiology

## Abstract

**Abstract:**

**Objectives:**

This study aimed to investigate the relationship between left ventricular (LV) remodelling and systolic function in high-gradient severe aortic stenosis (AS) using cardiovascular magnetic resonance (CMR) imaging. Patients with low ejection fraction (left ventricular ejection fraction (LVEF) <50%) high-gradient severe AS (low ejection fraction high-gradient (LEF-HG)), that is, afterload mismatch, were compared with patients with normal ejection fraction high-gradient (NEF-HG) severe AS. The hypothesis of insufficient left ventricular hypertrophy (LVH) leading to high wall stress as the mechanism of systolic dysfunction in afterload mismatch was tested.

**Design:**

This was a cross-sectional study design.

**Setting:**

The study was performed at a single tertiary academic hospital in the Western Cape, South Africa.

**Participants:**

A total of 48 participants with high-gradient (mean gradient ≥40 mm Hg) severe AS (aortic valve area (AVA) <1.0 cm^2^) were consecutively recruited. Of 48 participants, 24 (50%) had LEF-HG AS (mean AVA 0.52±0.18 cm^2^, mean gradient 54 (21) mm Hg, mean LVEF 29±11%). The remainder of the cohort had NEF-HG AS (mean AVA 0.70±0.17 cm^2^, mean gradient 53 (25) mm Hg, LVEF 64±14%).

**Results:**

End-systolic wall stress (ESWS) was higher in the LEF-HG group (265.3±83.5 vs 122.8±54.7×10^3^ dynes/cm^2^; p<0.0001) and associated significantly with LVEF. The degree of LVH measured by wall thickness (13.0 vs 13.0 mm, p>0.99) was identical between the groups. The LV mass index was numerically higher in the LEF-HG group (101.9±37.2 vs 77.6±22.2 g/m^2^) but this was not statistically significant (p=0.91). The LEF-HG AS group demonstrated significant LV cavity dilation (LV End-Diastolic Volume Index 124.9±29.9 vs 78.4±16.0 mL/m^2^, p<0.0001). Elevated ESWS was associated with smaller valve areas and cavity dilation but not with LVH.

**Conclusion:**

The reduced LVEF in afterload mismatch was associated with high ESWS as previously suggested. However, insufficient concentric LVH was not observed in LEF-HG AS and therefore not associated with high ESWS. LV cavity dilation and smaller valve areas were instead associated with the elevated ESWS in afterload mismatch, which may represent the decompensated stage of high-gradient severe AS.

**STRENGTHS AND LIMITATIONS OF THIS STUDY**

- Used cardiovascular magnetic resonance (CMR) imaging, which is the gold standard, to assess left ventricular (LV) morphology, volumes, function and geometry in patients with high-gradient severe aortic stenosis (AS) (comparing low ejection fraction high-gradient AS to normal ejection fraction high-gradient AS).
- End-systolic wall stress (ESWS) as a measure of afterload was estimated non-invasively using CMR. The relationships between LV geometry, function and ESWS were evaluated using univariate and multivariate regression analyses to better understand the mechanism(s) underlying the reduced left ventricular ejection fraction in afterload mismatch.
- There was a single-centre cross-sectional study design thus limiting the ability to define causation between geometric remodelling and functional status.

## Introduction

 Afterload mismatch is a unique yet critically understudied haemodynamic scenario in severe aortic stenosis (AS). For most patients with classic high-gradient severe AS, the left ventricle (LV) undergoes compensatory hypertrophy (LVH) with preservation of the LV ejection fraction (LVEF).^1^,^3^ The term ‘afterload mismatch’ suggests a scenario where a severe elevation in afterload is not matched by a similar hypertrophic response leading to persistently elevated wall stress and therefore afterload.^3 4^ Afterload mismatch is recognised clinically in patients with severe AS when LVEF is reduced (<50%) but there is maintenance of a high gradient in the severe range suggesting that LV pressure generation is good but muscle fibre shortening is impaired based on excessively elevated afterload.^5^ This, and the often immediate LVEF normalisation post aortic valve replacement (AVR),^6^ implies that the reduction in LV performance is mediated through mechanisms other than intrinsic contractile dysfunction.^4^,^7^ These mechanisms remain incompletely explored.

The hypothesis that myocardial fibrosis is the driving mechanism of systolic dysfunction in high-gradient severe AS was previously tested by our team.^8^ High end-systolic wall stress (ESWS) rather than myocardial fibrosis was, however, found to be significantly associated with LVEF.^8^ It is well known that LV performance (LVEF) is related to myocardial contractility but also to LV geometry/remodelling and the loading conditions experienced by the LV.^9^ Existing evidence, based on transthoracic echocardiography (TTE) and invasive cardiac catheterisation, suggests that the reduced LVEF in afterload mismatch results from insufficient LVH.^4 6 7^ Governed by the Law of Laplace, insufficient LVH results in failure to normalise wall stress and a consequent decline in LV performance (LVEF).^4 7^

The Laplace equation has traditionally been used to characterise LV wall stress which, in essence, reflects the afterload that the contracting ventricle (through fibre shortening) must overcome for adequate LV ejection to occur.^4 6 7^ Assuming a constant LV pressure, the two determinants of LV wall stress are geometric in nature: LV wall thickness and LV diameter.^4 6 7^ Although LVH severity is represented in the Laplace equation by wall thickness, another, more accurate measure of LVH which better assesses the increase in sarcomere number is LV mass (LVM). Alterations in LVM in afterload mismatch have previously been reported in a limited number of studies using TTE and cardiovascular magnetic resonance (CMR) imaging but their relationship with ESWS has yet to be systematically evaluated.^5 10^ Furthermore, whether changes in the other major geometric determinant of wall stress, that is, LV diameter, more accurately characterised as LV volume, is present and associated with high ESWS/reduced LVEF in high-gradient severe AS remains unexplored.

Cardiovascular MRI is the gold standard imaging technique for the evaluation of LV geometry, mass, volume and function.^11^ Unlike TTE and invasive catheterisation, the entire LV can be imaged thus avoiding geometric assumptions.^11^ This, together with the high contrast resolution achieved, significantly improves the accuracy and precision with which adaptive LV remodelling is assessed. Using CMR, six geometric patterns of LV adaptation have previously been described in moderate and severe AS.^12^ How these patterns relate to LV remodelling in the context of afterload mismatch needs to be explored and critically, whether the LVH response is in fact insufficient in this population as is currently suggested. We therefore used CMR to characterise the LV in afterload mismatch and compared LV remodelling in patients with afterload mismatch to that of patients with high-gradient severe AS and preserved LVEF. We hypothesised that the LVH response in afterload mismatch is not insufficient and rather, that if excessive wall stress is present, it is related primarily to adverse LV geometric remodelling in the form of LV dilation.

This study therefore contributes an assessment of LV geometric remodelling in high-gradient severe AS with afterload mismatch using the gold standard imaging modality of CMR adding to the limited body of existing knowledge, and re-explores the interaction between LV geometry and ESWS. Understanding the differences in LV remodelling between the two groups may provide valuable insight into the mechanisms underlying the poor LV performance in afterload mismatch. In clinical practice, this may enable the identification of imaging features that signify a decline towards poor LV performance in severe AS, prompting decision-making for earlier AVR.

## Methods

### Study design

Between June 2021 and July 2024, 48 participants with high-gradient (mean transvalvular gradient ≥40 mm Hg) severe AS (aortic valve area (AVA) <1.0 cm^2^ and peak velocity ≥4 m/s) were consecutively enrolled for CMR from a tertiary academic centre in the Western Cape, South Africa. Patients were grouped according to LVEF above or below 50%, that is, those with an LVEF <50% were considered as afterload mismatch (low ejection fraction high-gradient (LEF-HG)) and those with an LVEF ≥50% were considered as the normal LVEF high-gradient group (normal ejection fraction high-gradient (NEF-HG)). Exclusion criteria included significant coronary artery disease (stenosis of >50%) confirmed by coronary angiography, other haemodynamically significant valve lesions, comorbid structural heart diseases (including cardiac amyloidosis) and contraindications to CMR. At enrolment, a detailed history including presenting symptoms, New York Heart Association status, comorbidities and smoking history were recorded. A clinical examination was performed where resting blood pressure, anthropometry and heart failure status were recorded. All participants underwent cardiovascular imaging using TTE and CMR.

### Transthoracic echocardiography

TTE was performed by an experienced echocardiographer in our departmental echocardiography centre. All measurements were performed in accordance with the British Society of Echocardiography guidelines pertaining to the standardised protocol for imaging acquisition and measurement relating to the relevant structures.^13^ Peak transaortic velocity was acquired using continuous wave (CW) Doppler and the modified Bernoulli equation was used to derive the peak and mean pressure gradients.^14^ Standalone CW Doppler-only probes were used from suprasternal and supraclavicular, as well as right parasternal, apical and subcostal windows to ensure maximum aortic valve (AV) velocities were acquired. The left ventricular outflow tract (LVOT) diameter and velocity-time integral were measured just below the level of the AV cusp insertion and the former, at peak systole, coinciding with maximal AV leaflet excursion. Using pulse wave (PW) Doppler, the Doppler region of interest was optimised manually to ensure a crisp PW trace outline, without edge smearing, as close to the valve as possible from a five-chamber or three-chamber orientation, whichever afforded the best alignment with LVOT flow. The AVA was derived using the continuity equation.^14^

### CMR image acquisition

Cardiovascular MRI was performed on a 1.5 Tesla scanner (Siemens, Magnetom Aera, Erlangen, Germany) using a standardised, guideline-based protocol.^15^ Cardiac-gated, breath-held cine imaging was performed using a balanced steady state-free precession sequence. Long axis two-chamber, three-chamber and four-chamber views, as well as a short axis stack from base to apex were acquired. Typical cine image acquisition parameters included an 8 mm slice thickness with a 2 mm gap where applicable, 25 phases, TR 35–45 ms, TE 1.2 ms, matrix size 156×192 with voxel size ±1.9×1.9 mm. Through-plane phase contrast flow at the level of the AV tips was acquired in accordance with guideline recommendations.^15^ Typical acquisition parameters included a 5 mm slice thickness, 50 phases, TR 26 ms, TE 3.71 ms, matrix size 208×256 with voxel size ±1.6×1.6 mm.

### CMR image analysis

Image analysis was performed using CVI42 (V.5.13.7, Circle Cardiovascular Imaging, Calgary, Canada). Wall thickness was measured in the mid-ventricular septum of the short axis cine stack ensuring that the segment measured was perfectly perpendicular to the long axis of the septum. Endocardial and epicardial contours were semi-automatically drawn in the short axis stack at end-diastole and end-systole, for the evaluation of LV end-diastolic volume (LVEDV), LV end-systolic volume (LVESV), LV stroke volume (LVSV), LVEF and LVM. Papillary muscles were manually excluded from the blood pool and were included in the LVM calculation as per consensus recommendations.^16^ The LV volumes/mass were indexed to the Mosteller derived body surface area and are denoted by the letter ‘i’. Transaortic flow rate was calculated by dividing the LVSV by the systolic ejection time derived from aortic phase contrast flow curves.

### Geometric LV characterisation

LV geometry was assessed using CMR-derived parameters that included LV wall thickness, LVM and LV volumes. Based on the work of Dweck *et al* who defined six geometric patterns in AS, we used LVM indexed (LVMi), LVEDV indexed (LVEDVi), mass/volume ratio and LVEF to categorise participants in our cohort into one of the six predefined categories.^12^ These predefined categories included a normal geometric pattern, concentric remodelling, asymmetric remodelling, concentric hypertrophy, asymmetric hypertrophy and LV decompensation.^12^ Sphericity index was calculated using the equation described by Ambale-Venkatesh *et al.*^17^

### Non-invasive meridional end-systolic wall stress analysis

Meridional ESWS was calculated non-invasively using the Laplace-derived equation previously described and validated against invasive measurements by Reichek *et al.*^18^ The LV end-systolic internal diameter and wall thickness were measured in a basal short axis cine slice that excluded the LVOT as illustrated by Carter-Storch *et al.*^19^ The recommendation from Reichek *et al* was to use systolic blood pressure (SBP) (cuff pressure) as an invasively-validated surrogate for LV pressure. This validation however, was performed in a non-AS cohort.^18^ To account for high transvalvular pressure gradients in this study, LV pressure was estimated using the sum of SBP measured at the time of CMR and the mean transvalvular pressure gradient derived on TTE.^20^

Meridional ESWS was then calculated using the formula below:


$$Meridonial\;ESWS=\frac{0.334xPxLVIDs}{WTsx(1+\frac{WTs}{LVIDs})}$$

Where:

*P=SBP+mean transaortic pressure gradient.*

*LVIDs=end-systolic LV internal diameter.*

*WTs=end-systolic wall thickness.*

In a subset of patients (n=17), invasive cardiac catheterisation was performed (methodology and results previously reported by Liebenberg *et al*).^5^ Our non-invasive measurements were correlated with the invasive measurements of ESWS by Liebenberg *et al* to establish the robustness of our non-invasive method.

### Statistical analysis

GraphPad Prism (V.10.0.2, GraphPad, Boston, USA) and SPSS (V.30.0, New York, IBM) were used for statistical analysis. To detect mean volumetric and mass differences of at least 10 mL and 10 g, respectively, with a power of 95% at an alpha of 5%, a minimum sample size of 12 (per study group) was required and met in this study. Categorical variables are presented as absolute numbers and percentages and were compared using the χ^2^ test. The Shapiro-Wilk test was used for normality testing in view of the sample size. Normally distributed continuous variables are reported as mean±SD and were compared using the Student’s t-test. Non-normally distributed continuous variables are reported as median and IQR and compared using the Mann-Whitney U test. A p value<0.05 was considered statistically significant. To control for multiple testing and the family-wise error rate, the Welch’s analysis of variance test was performed for CMR parameters. Post hoc p value corrections were then derived using the Dunnett’s T3 test. Adjusted p values are reported where applicable. Simple linear (univariate) regression analyses were performed to measure the strength of associations between relevant variables of LV remodelling and LVEF/ESWS. To confirm independence of association between parameters of LV remodelling and LVEF/ESWS, multivariate regression was also performed to control for known confounding variables including age, sex, body mass index (BMI), SBP and AVA. Where necessary, weighted least squares regression was performed to correct for heteroscedasticity of the data. All other assumptions were fulfilled for the multivariate regression models.

### Patient and public involvement

There were no patient/public involvement in the study design or conduct.

## Results

### Study population

The total cohort that met the inclusion criteria comprised 48 participants with high-gradient severe AS, including 31 (65%) females and 17 (35%) males (table 1). Half the cohort (24 (50%)) had LEF-HG AS with a mean LVEF of 29±11%. The remaining half (24 (50%)) had preserved systolic function (NEF-HG AS) with a mean LVEF of 64±14%. The mean AVA was significantly smaller in those with LEF-HG AS (0.52±0.18 vs 0.70±0.17 cm^2^) (table 1). The median mean gradients were similar between the groups (table 1). However, this finding was observed within the context of a lower flow rate in the LEF-HG group (table 2). While most baseline characteristics were statistically similar between the groups, patients with LEF-HG AS were more likely to have dyspnoea on presentation and also trended toward a higher rate of heart failure at the time of recruitment (table 1). The total cohort had a high prevalence of hypertension with significantly higher systolic blood pressure observed in the NEF-HG group (table 1). There were no cases of concomitant cardiac amyloidosis in either group (table 1).

**Table 1:** Baseline characteristics of the study population

Parameter	LEF-HG (n=24)	NEF-HG (n=24)	P value
Age (years)	58±13	63±8	0.09
Sex			
Female	16 (67%)	15 (63%)	0.76
Male	8 (33%)	9 (37%)	0.76
Chest pain	11 (46%)	11 (46%)	>0.99
Dyspnoea	24 (100%)	15 (63%)	**0.0009**
Syncope	7 (29%)	7 (29%)	>0.99
Fatigue	19 (79%)	17 (71%)	0.51
Hypertension	14 (58%)	19 (79%)	0.12
Diabetes mellitus	4 (17%)	8 (33%)	0.18
Dyslipidaemia	5 (21%)	8 (33%)	0.33
Past/present smoker	10 (42%)	12 (50%)	0.56
Heart failure on current admission	14 (58%)	8 (33%)	0.08
Heart rate (beats per minute)	82±14	73±13	**0.02**
Resting SBP (mm Hg)	114±20	134±18	**0.0006**
Resting DBP (mm Hg)	72±8	74±13	0.54
Body mass index (kg/m^2^)	28±7	31±7	0.13
Mean AVA (cm^2^)	0.52±0.18	0.70±0.17	**0.001**
Mean gradient (mm Hg)	54 (21)	53 (25)	0.86
Peak gradient (mm Hg)	89 (36)	80 (37)	0.54
AS aetiology			
Calcific degenerative AS	9 (38%)	10 (42%)	0.77
Rheumatic AS	7 (29%)	7 (29%)	>0.99
Bicuspid AS	8 (33%)	7 (29%)	0.76
Incidental amyloidosis*	0 (0)	0 (0)	N/A

Biological sex (male/female/intersex) ascertained by self-report.

Statistically significant p values (ie, <0.05) are illustrated in bold.

For continuous variables, p values were derived using the Student’s t-test or Mann-Whitney U test based on normal or skewed distributions, respectively.

For categorical variables, p values were derived using the χ2 test.

* Incidental amyloidosis as per CMR features.

AS, aortic stenosis; AVA, aortic valve area; CMR, cardiovascular magnetic resonance; DBP, diastolic blood pressure; LEF-HG, low ejection fraction high-gradient; NEF-HG, normal ejection fraction high-gradient; SBP, systolic blood pressure.

**Table 2:** Comparison of LV and LA morphological and functional characteristics in patients with LEF-HG AS and those with NEF-HG AS

Parameter	LEF-HG (n=24)	NEF-HG (n=24)	Adjusted p value
LVEDV (mL)	226.0±49.7	150.2±29.6	**0.0001**
LVEDVi (mL/m^2^)	124.9±29.9	78.4±16.0	**<0.0001**
LVESV (mL)	164.1±43.9	53.6±18.4	**<0.0001**
LVESVi (mL/m^2^)	90.5±24.2	28.0±10.1	**<0.0001**
LVSV (mL)	61.9±20.8	96.7±22.8	**0.001**
LVSVi (mL/m^2^)	34.5±13.5	50.3±11.8	0.05
Flow rate (mL/s)	184.3±72.2	271.2±64.0	0.06
LVEF (%)	29±11	64±14	**<0.0001**
LVM (g)	184.6±60.7	150.4±48.5	0.99
LVMi (g/m^2^)	101.9±37.2	77.6±22.2	0.91
Wall thickness using chords method (mm)			
Basal	9.2 (2.8)	9.8 (2.4)	>0.99
Mid-ventricular	8.8 (2.0)	8.9 (1.5)	>0.99
Apical	6.5 (3.1)	6.3 (2.3)	>0.99
Wall thickness (single thickest segment) (mm)	13.0 (2.8)	13.0 (2.0)	>0.99
Sphericity Index	0.5±0.1	0.5±0.1	>0.99
M/V ratio	0.8 (0.2)	0.9 (0.2)	0.99
h/r	0.4±0.1	0.5±0.1	0.81
Meridional ESWS (×10^3^ dynes/cm^2^)	265.3±83.5	122.8±54.7	**<0.0001**
LA diameter (cm)	4.0±0.5	3.4±0.7	**0.003**
LA area (cm^2^)	26.4±6.8	24.3±5.7	0.26
LAVi (mL/m^2^)	49.1 (18.0)	40.0 (14.6)	0.06

Statistically significant p values (<0.05) are illustrated in bold.

Differences were tested using the Welch’s ANOVA test due to heterogeneity of variances. Adjusted p values derived from the Dunnett’s T3 test are reported.

The letter ‘I’ denotes parameter indexed for body surface area.

ANOVA, analysis of variance; AS, aortic stenosis; ESWS, end-systolic wall stress; h/r, wall thickness to chamber radius ratio; LA, left atrium; LAVi, left atrial volume indexed for body surface area; LEF-HG, low ejection fraction high-gradient; LV, left ventricle; LVEDV, LV-end-diastolic volume; LVEF, LV ejection fraction; LVESV, LV end-systolic volume; LVM, LV mass; LVMi, left ventricular mass indexed for body surface area; LVSV, LV stroke volume; M/V, mass-volume ratio; NEF-HG, normal ejection fraction high-gradient.

### Left ventricular morphology and function

The end-diastolic wall thickness was increased and identical between the two groups (13.0 (2.8) vs 13.0 (2.0) mm). In a multivariate regression analysis adjusting for age, sex, BMI, SBP and AVA, sex was found to be significantly associated with wall thickness (online supplemental table 1). Using LVM/LVMi indices, both groups of patients had evidence of LVH according to the reference ranges defined by the Society of Cardiovascular Magnetic Resonance (SCMR).^21^ Although the absolute LVMi was higher in the LEF-HG group (mean LVMi=101.9 ± 37.2 vs 77.6±22.2 g/m^2^), the difference was not statistically significant (p=0.91) (table 2). Significant cavity dilation was present in the LEF-HG AS group with a mean LVEDVi of 124.9±29.9 mL/m^2^ (table 2). Cavity dilation was not a feature in those with NEF-HG AS (mean LVEDVi 78.4±16.0 mL/m^2^) (table 2). The mass/volume (M/V) and wall thickness/chamber radius (h/r) ratios were statistically similar between groups (table 2). The LVSVi measured below the normal limit for those with LEF-HG AS (34.5±13.5 mL/m^2^) and the absolute stroke volume (LVSV) was significantly lower compared with those with NEF-HG AS (table 2). A lower flow rate was also found in those with LEF-HG AS (184.3±72.2 vs 271.2±64.0 mL/s; p=0.06). The prevalence of pleural and pericardial effusions was higher in the LEF-HG group (71% vs 13% and 50% vs 8%, respectively) reflecting the impact of systolic dysfunction in these patients.

### Left ventricular geometric patterns

Based on increased LVMi, increased LVEDVi and reduced LVEF, 22 (92%) patients with LEF-HG AS were categorised into the decompensated pattern of LV remodelling (table 3). The remaining 2 (8%) patients with LEF-HG AS fitted into none of the categories. In those with NEF-HG AS, 9 (38%) demonstrated a normal geometric pattern, 7 (29%) demonstrated a concentric hypertrophy pattern and 8 (33%) fitted into none of the predefined categories (table 3). In the 10 patients who fitted into none of the predefined geometric patterns, an increased LVEDVi was present together with upper limit of normal to mildly increased LVMi and an M/V ratio of 0.9 or less (reduced LVEF was present in 2 (20%) of these patients). None of the patients demonstrated concentric remodelling, asymmetric remodelling or asymmetric hypertrophy.

**Table 3:** Frequency and distribution of remodelling patterns in patients with LEF-HG AS and NEF-HG AS

Category	LEF-HG (n=24)	NEF-HG (n=24)
Normal	0 (0%)	9 (38%)
Concentric remodelling	0 (0%)	0 (0%)
Asymmetric remodelling	0 (0%)	0 (0%)
Concentric hypertrophy	0 (0%)	7 (29%)
Asymmetric hypertrophy	0 (0%)	0 (0%)
LV decompensation	22 (92%)	0 (0%)
None	2 (8%)	8 (33%)

AS, aortic stenosis; LEF-HG, low ejection fraction high-gradient; LV, left ventricular; NEF-HG, normal ejection fraction high-gradient.

The maintenance of a conical LV shape despite LV dilation in the LEF-HG group was illustrated by a mean sphericity index of 0.5±0.1 in both groups of patients (table 2).

### Meridional end-systolic wall stress and relationship with LV geometry and systolic dysfunction

Non-invasive ESWS derived by the method described in this paper was linearly associated with the gold standard method, that is, invasive cardiac catheterisation derived ESWS (standardised β coefficient=0.81, 95% CI 1.46 to 3.40, p<0.001) (online supplemental table 2 and online supplemental figure 1). Meridional ESWS was significantly higher in the LEF-HG AS group (265.3±83.5 vs 122.8±54.7; p<0.0001) (table 2). A strong inverse linear relationship was observed between ESWS and LVEF (standardised β coefficient −0.75 with 95% CI −0.19 to −0.11 and p<0.001) (table 4). In a multivariate analysis adjusting for age, sex, BMI, SBP and AVA, ESWS was found to be independently associated with LVEF (standardised β coefficient −0.66 with 95% CI −0.18 to −0.10 and p<0.001) (table 4).

**Table 4:** Univariate and multivariate analyses for associations with LVEF

Univariate analysis for LVEF				
Variable	Unstandardised β coefficient	95% CI	Standardised β coefficient	P value
Age (years)	0.61	0.08 to 1.13	0.33	**0.02**
Sex	−0.42	−13.02 to 12.17	−0.10	0.95
BMI (kg/m^2^)	0.56	−0.30 to 1.42	0.19	0.19
SBP (mm Hg)	0.48	0.23 to 0.73	0.49	**<0.001**
AVA (cm^2^)	46.58	19.15 to 74.00	0.45	**0.001**
ESWS (×10^3^dynes/cm^2^)	−0.15	−0.19 to −0.11	−0.75	**<0.001**

Multivariable analysis for LVEF				
Variable	Unstandardised β coefficient	95% CI	Standardised β coefficient	P value
Age (years)	0.35	−0.03 to 0.72	0.19	0.07
Sex	3.06	−5.39 to 11.50	0.07	0.47
BMI (kg/m^2^)	−0.44	−0.64 to 0.56	−0.02	0.88
SBP (mm Hg)	0.18	−0.03 to 0.39	0.19	0.10
AVA (cm^2^)	8.19	−13.44 to 29.81	0.07	0.45
ESWS (×10^3^dynes/cm^2^)	−0.13	−0.18 to −0.10	−0.66	**<0.001**

Biological sex (male/female/intersex) ascertained by self-report.

Multivariate model performance/significance: R=0.82, R2=0.67, *F*(6,41)=14.14 with p<0.001.

Significant associations (p<0.05) illustrated in bold.

AVA, aortic valve area; BMI, body mass index; ESWS, end-systolic wall stress; LVEF, left ventricular ejection fraction; SBP, systolic blood pressure.

In a univariate analysis of LV geometric associations with ESWS, SBP, AVA, LV cavity size and h/r ratio were found to interact significantly with ESWS (table 5). At multivariate analysis, AVA and LV cavity size remained significant and LVMi was also found to have a significant association with ESWS (table 5). Cavity size whether by two-dimensional measurement (LV internal diameter in diastole, ie, LVIDd) or by volumetric assessment (LVEDVi), had the strongest association with ESWS (LVIDd: standardised β coefficient of 0.52 with 95% CI 3.79 to 9.61 and p<0.001; LVEDVi: standardised β coefficient of 0.66 with 95% CI 1.43 to 3.19 and p<0.001) (table 5).

**Table 5:** Univariate and multivariate analyses for LV geometric associations with ESWS

Univariate analysis for ESWS				
Variable	Unstandardised β coefficient	95% CI	Standardised β coefficient	P value
Age (years)	−0.79	−3.49 to 1.91	−0.09	0.56
Sex	36.05	−24.58 to 96.69	0.17	0.24
SBP (mm Hg)	−1.45	−2.79 to −0.12	−0.31	**0.03**
AVA (cm^2^)	−211.31	−347.72 to −74.89	−0.42	**0.003**
LVIDd (mm)	5.30	2.20 to 8.39	0.45	**0.001**
WTd	−5.74	−17.77 to 6.29	−0.14	0.34
h/r ratio	−425.80	−685.74 to −165.87	−0.44	**0.002**
LVEDVi (mL/m^2^)	1.81	1.10 to 2.52	0.60	**<0.001**
LVMi (g/m^2^)	0.43	−0.47 to 1.34	0.14	0.34

Multivariable analysis for ESWS: Model A				
Variable	Unstandardised β coefficient	95% CI	Standardised β coefficient	P value
Age (years)	0.16	−2.06 to 2.38	0.02	0.89
Sex	49.12	−1.45 to 99.68	0.26	0.06
SBP (mm Hg)	−1.03	−2.15 to 0.08	−0.23	0.07
AVA (cm^2^)	−123.20	−228.84 to −17.56	−0.27	**0.02**
LVIDd (mm)	6.70	3.79 to 9.61	0.52	**<0.001**
WTd	−4.87	−13.33 to 3.59	−0.15	0.23

Multivariable analysis for ESWS: Model B				
Variable	Unstandardised β coefficient	95% CI	Standardised β coefficient	P value
Age (years)	0.16	−1.76 to 2.08	0.02	0.87
Sex	36.77	−6.79 to 80.33	0.21	0.10
SBP (mm Hg)	−0.72	−1.70 to 0.26	−0.17	0.15
AVA (cm^2^)	−104.69	−205.37 to −4.01	−0.23	**0.04**
LVEDVi (mL/m^2^)	2.31	1.43 to 3.19	0.66	**<0.001**
LVMi (g/m^2^)	−0.88	−1.71 to −0.06	−0.28	**0.04**

Biological sex (male/female/intersex) ascertained by self-report.

Multivariate model A performance/significance: R=0.76, R2=0.58, *F*(6,41)=9.23 with p<0.001.

Multivariate model B performance/significance: R=0.80, R2=0.63, *F*(6,41)=11.76 with p<0.01.

Weighted least squares regression performed to correct for heteroscedasticity in models A and B.

Significant associations (p<0.05) illustrated in bold.

AVA, aortic valve area; ESWS, end-systolic wall stress; LVEDVi, left ventricular end-diastolic volume indexed for body surface area; LVIDd, left ventricular internal diameter in diastole; LVMi, left ventricular mass indexed for body surface area; h/r ratio, end-diastolic wall thickness to LV radius ratio; SBP, systolic blood pressure; WTd, wall thickness in diastole.

## Discussion

This study demonstrates that LEF-HG AS is characterised by adaptive LVH comparable to that of patients with NEF-HG AS in terms of wall thickness and cardiac mass (LVM) added. This contradicts the idea that a poor LVH response lies at the heart of afterload mismatch. Elevated ESWS, illustrated to be closely related to systolic dysfunction, was associated primarily with LV cavity dilation observed in the LEF-HG group. Furthermore, smaller valve areas in this group suggested the presence of more advanced AS disease and this may support the hypothesis of LV dilation representing decompensation in high-gradient severe AS.

### Adaptive LVH, wall stress and LVEF in afterload mismatch

Earlier work by Gunther *et al*, demonstrated that LVEF in high-gradient severe AS was near-perfectly inversely related to invasively measured circumferential midwall ESWS.^4^ This key finding was replicated in our study. According to the Law of Laplace, a major determinant of wall stress is wall thickness.^4^ In the work of Gunther *et al*, those patients with AS found to have a reduced LVEF with excessively high wall stress were found to have minimal (or even no) LVH (ranging from 0.9 to 1.1 cm).^4^ Where LVEF was reduced but wall stress normalised, concentric LVH was present (ranging from 1.29 to 1.35 cm).^4^ This evidence led to the hypothesis that the reduced LVEF in afterload mismatch was driven by high wall stress resulting from a failure of the LV to undergo a sufficient degree of concentric LVH. In our study, the entire cohort met criteria for increased wall thickness/LVM and for those with LEF-HG AS, the degree/extent of concentric LVH was comparable to that of patients with NEF-HG AS. In the regression analyses, wall thickness was not found to interact significantly with ESWS. Together, these findings contradict the historical hypothesis that afterload mismatch is driven by insufficient concentric LVH. Although sex was significantly associated with wall thickness in this study, the female/male distributions were near identical between the study groups with a difference of only 4%. Both technical and physiological explanations for the difference in findings exist.

The difference in findings may have resulted from the different techniques used to evaluate LVH. Earlier work performed prior to the advent of CMR relied on invasive cardiac catheterisation and TTE for LVH evaluation. It is now widely accepted that CMR is the most accurate and precise tool for the assessment of LV remodelling including the evaluation of LVH, and this is reflected in its selection as the imaging gold standard for the morphological and volumetric/functional evaluation of the LV.^11^ Another consideration is that existing evidence on LV remodelling in afterload mismatch is based on work performed in Western/European populations. In other high afterload conditions such as systemic arterial hypertension, ancestry-driven variation in the LVH response to a high afterload is well documented.^22^,^27^ Given the paucity of AS studies in the African context, the impact of ancestry on LV remodelling in our cohort is unknown highlighting the need for further studies in Africa.

### LV dilation and elevated wall stress in afterload mismatch

Wall stress in our cohort of patients with LEF-HG AS was significantly higher than in the NEF-HG AS group despite them undergoing a comparable degree of concentric LVH. This highlights the importance of evaluating the remaining determinants of wall stress such as chamber radius. Using both the two-dimensional LV diameter and three-dimensional LV volume, significant LV dilation was observed in the LEF-HG group. While LV dilation is not typically observed in isolated pressure overload conditions, the finding of LV dilation has been made in other studies of LEF-HG AS using TTE and CMR.^5 10^ In one of these studies, the LEF-HG AS group was also found to have LVH and elevated wall stress measured on invasive cardiac catheterisation.^5^ In our univariate and multivariate regression analyses, the LV size was shown to have a significant association with ESWS. Based on the Laplace Law, concentric LVH is required for wall stress normalisation but the benefit of this in our cohort appears to have been outweighed by the co-presence of significant LV dilation.

A limitation of this study was its cross-sectional design which challenges our ability to define the temporal relationship between LV dilation and ESWS. Whether some patients were predestined towards LV dilation as an initial adaptive response to pressure overload or whether LV dilation is a decompensatory consequence to long-standing pressure overload that has outweighed adaptive concentric LVH is therefore unknown. An argument could be made for the latter based on the observations that the severity of AS was worse in the LEF-HG group and that valve area covariates with LV dilation as a predictor of ESWS in our multivariate regression analyses.

### Afterload mismatch may represent a more advanced stage of severe AS

The place of afterload mismatch in the natural history of severe AS has not been clearly defined. In our cohort of patients with LEF-HG AS, the valve areas were significantly smaller than in the NEF-HG group suggesting more severe degrees of valve stenosis. In addition, the mean gradients were found to be similar between the groups despite significantly lower LVSV and flow rates in the LEF-HG group. A higher wall stress in the LEF-HG AS group together with a significant inverse relationship observed between ESWS and AVA suggests that high ESWS in afterload mismatch was at least in part related to excessively high afterload rather than adverse geometric remodelling alone. Taken together, an argument can be made that afterload mismatch may represent a more advanced stage in the natural progression of severe AS. In further support of this, 20% of the cohort were not categorised into any of the remodelling patterns described by Dweck *et al.*^12^ On closer inspection, the ventricles in this subgroup were found to be heavy (in terms of LVMi) with upper limit of normal to mildly dilated cavity sizes essentially resembling the morphology of the LV in LEF-HG AS but with preserved LVEF. Whether these patients were in a transitory period from NEF-HG AS to the LEF-HG state is unknown and could not be determined by the cross-sectional nature of this study. While longitudinal follow-up of this group would have been useful, it was not ethically possible given that indications for valve replacement were already present.

The hypothesis that afterload mismatch represents an advanced stage of classic high-gradient severe AS is shared by other scholars in the field.^10 28^ In a large imaging-based study by Puls *et al*, those with LEF-HG AS were found to have dilated ventricles with increased LVM and smaller valve areas when assessed through TTE, similar to the findings of our study.^10^ They were also found to have a higher myocardial fibrosis burden and worse clinical outcomes compared with patients with NEF-HG AS.^10^ While it is plausible that afterload mismatch represents a more advanced stage than classic high-gradient severe AS, it may not represent the true end-stage of the disease. Longitudinal studies that have evaluated the impact of AVR on clinical outcomes have demonstrated worse outcomes in patients with low-gradient severe AS and systolic dysfunction than in patients with LEF-HG AS.^10 28 29^ Afterload mismatch, therefore, may represent an intermediate state where NEF-HG severe AS transitions towards low-gradient (LEF-LG) severe AS. This may serve as a critical window period to intervene before irreversible LV damage and progression to the poorly prognostic low-gradient state occurs.

## Limitations

This is a single-centre study that was limited by a small sample size. However, given the high contrast resolution of CMR resulting in low interobserver and intraobserver variability enables a significant reduction in sample size requirements to demonstrate differences between groups compared with other imaging modalities. A sample size calculation suggested recruited numbers were adequate to answer the posed question. Also, afterload mismatch represents a small proportion of patients with severe AS and reassuringly, the distribution of data followed a normal Gaussian distribution for the majority of parameters evaluated.

Readers were blinded to case categories. However, it is difficult to truly blind MRI readers to the group being assessed as the LVEF difference between the groups was large and therefore lower LVEF would have been identifiable as afterload mismatch by the reader. However, the high contrast resolution offered by CMR limits the uncertainty of measurement edges and improves measurement reliability. The large differences identified between groups also makes bias a less likely cause of the findings. Our group’s interstudy reliability has previously been published in pathology assessing LV remodelling and specifically, LVH.^30^

Finally, being a cross-sectional study, the ability to separate cause from effect must be taken into consideration. While important associations were demonstrated in this study, correlation is not equivalent to causation. Larger longitudinal studies designed to interrogate afterload mismatch are still needed.

## Conclusion

This study used CMR imaging to evaluate LV remodelling in high-gradient severe AS and its role on reduced LVEF in afterload mismatch. In keeping with prior observations, the reduced LVEF in high-gradient severe AS was associated with high ESWS. However, insufficient LVH was not present in our cohort of afterload mismatch patients as previously suggested. Smaller valve areas and LV dilation were observed in afterload mismatch and rather than LVH, these variables were significantly co-associated with the high wall stress. Afterload mismatch representing a decompensated stage in the natural history of high-gradient severe AS requires further investigation in a longitudinal study design.

## Supplementary material

10.1136/bmjopen-2026-118642 online supplemental file 1

## Data Availability

All data relevant to the study are included in the article or uploaded as supplementary information.

## References

[1] Lancellotti P (2012). Grading aortic stenosis severity when the flow modifies the gradientvalve area correlation. Cardiovasc Diagn Ther.

[2] Kwon S, Gopal A, Magnusson P (2019). Aortic stenosis – current perspectives.

[3] Carabello BA (2021). The Pathophysiology of Afterload Mismatch and Ventricular Hypertrophy. *Structural Heart*.

[4] Gunther S, Grossman W (1979). Determinants of ventricular function in pressure-overload hypertrophy in man. Circulation.

[5] Liebenberg J, Doubell A, Steyn J (2023). Exploring the mechanisms responsible for reduced systolic function in high-gradient aortic stenosis. Heart.

[6] Carabello BA, Green LH, Grossman W (1980). Hemodynamic determinants of prognosis of aortic valve replacement in critical aortic stenosis and advanced congestive heart failure. Circulation.

[7] Strauer BE (1979). Myocardial oxygen consumption in chronic heart disease: role of wall stress, hypertrophy and coronary reserve. Am J Cardiol.

[8] Rajah MR, Doubell AF, Herbst PG (2025). High afterload rather than myocardial fibrosis predicts reduced ejection fraction in severe aortic stenosis with afterload mismatch. Open Heart.

[9] Mewton N, Opdahl A, Choi E-Y (2013). Left ventricular global function index by magnetic resonance imaging--a novel marker for assessment of cardiac performance for the prediction of cardiovascular events: the multi-ethnic study of atherosclerosis. Hypertension.

[10] Puls M, Beuthner BE, Topci R (2020). Impact of myocardial fibrosis on left ventricular remodelling, recovery, and outcome after transcatheter aortic valve implantation in different haemodynamic subtypes of severe aortic stenosis. Eur Heart J.

[11] Suinesiaputra A, Bluemke DA, Cowan BR (2015). Quantification of LV function and mass by cardiovascular magnetic resonance: multi-center variability and consensus contours. J Cardiovasc Magn Reson.

[12] Dweck MR, Joshi S, Murigu T (2012). Left ventricular remodeling and hypertrophy in patients with aortic stenosis: insights from cardiovascular magnetic resonance. J Cardiovasc Magn Reson.

[13] Robinson S, Rana B, Oxborough D (2020). A practical guideline for performing a comprehensive transthoracic echocardiogram in adults: the British Society of Echocardiography minimum dataset. Echo Res Pract.

[14] Ring L, Shah BN, Bhattacharyya S (2021). Echocardiographic assessment of aortic stenosis: a practical guideline from the British Society of Echocardiography. Echo Res Pract.

[15] Kramer CM, Barkhausen J, Flamm SD (2013). Standardized cardiovascular magnetic resonance (CMR) protocols 2013 update. J Cardiovasc Magn Reson.

[16] Schulz-Menger J, Bluemke DA, Bremerich J (2020). Standardized image interpretation and post-processing in cardiovascular magnetic resonance - 2020 update. J Cardiovasc Magn Reson.

[17] Ambale-Venkatesh B, Yoneyama K, Sharma RK (2017). Left ventricular shape predicts different types of cardiovascular events in the general population. Heart.

[18] Reichek N, Wilson J, St John Sutton M (1982). Noninvasive determination of left ventricular end-systolic stress: validation of the method and initial application. Circulation.

[19] Carter-Storch R, Moller JE, Christensen NL (2019). End-systolic wall stress in aortic stenosis: comparing symptomatic and asymptomatic patients. Open Heart.

[20] Liebenberg J, Doubell A, Laubscher R (2023). In-silico modelling of the impact of hypertension on the mean transvalvular gradients in aortic stenosis. PLoS One.

[21] Kawel-Boehm N, Hetzel SJ, Ambale-Venkatesh B (2025). Society for Cardiovascular Magnetic Resonance reference values (“normal values”) in cardiovascular magnetic resonance: 2025 update. J Cardiovasc Magn Reson.

[22] Ahmad FS, Cai X, Kunkel K (2017). Racial/Ethnic Differences in Left Ventricular Structure and Function in Chronic Kidney Disease: The Chronic Renal Insufficiency Cohort. Am J Hypertens.

[23] Elghazaly H, McCracken C, Szabo L (2023). Characterizing the hypertensive cardiovascular phenotype in the UK Biobank. Eur Heart J Cardiovasc Imaging.

[24] Mohamed AT, Georgiopoulos G, Faconti L (2021). In-depth phenotyping by cardiovascular magnetic resonance uncovering differences between ethnic groups in hypertensive heart disease. Eur Heart J.

[25] Lewis AA, Ayers CR, Selvin E (2020). Racial Differences in Malignant Left Ventricular Hypertrophy and Incidence of Heart Failure: A Multicohort Study. Circulation.

[26] Drazner MH, Dries DL, Peshock RM (2005). Left ventricular hypertrophy is more prevalent in blacks than whites in the general population: the Dallas Heart Study. *Hypertension*.

[27] Fernandes-Silva MM, Shah AM, Hegde S (2017). Race-Related Differences in Left Ventricular Structural and Functional Remodeling in Response to Increased Afterload: The ARIC Study. JACC Heart Fail.

[28] Mutlak D, Aronson D, Lessick J (2010). Frequency, characteristics, and outcome of patients with aortic stenosis, left ventricular dysfunction, and high (versus low) trans-aortic pressure gradient. Isr Med Assoc J.

[29] Ternacle J, Faroux L, Alperi A (2021). Impact of Left-Ventricular Dysfunction in Patients With High- and Low- Gradient Severe Aortic Stenosis Following Transcatheter Aortic Valve Replacement. Can J Cardiol.

[30] Robbertse P-PS, Doubell AF, Steyn J (2023). Altered cardiac structure and function in newly diagnosed people living with HIV: a prospective cardiovascular magnetic resonance study after the initiation of antiretroviral treatment. Int J Cardiovasc Imaging.

