# Supplementary material for: Morphological and functional evaluation of the left ventricle in severe aortic stenosis with afterload mismatch: a South African single-centre, cross-sectional cardiovascular MRI-based study
Source: BMJ Open. 2026 Jun 24;16(6):e118642. doi: 10.1136/bmjopen-2026-118642 (PMC13295984; doi:10.1136/bmjopen-2026-118642)
Supplement: online supplemental file 1 [file bmjopen-16-6-s001.docx]

**Supplementary Section**

**Supplementary Table 1**

**Suppl. Table 1. Univariate and multivariate analyses for associations with end-diastolic wall thickness.**

| **Univariate Analysis for WTd** | | | | |
| --- | --- | --- | --- | --- |
| **Variable** | Unstandardized β Coefficient | 95% Confidence Interval | Standardized β Coefficient | p value |
| **Age (years)** | 0.00 | -0.07 to 0.07 | 0.00 | 0.99 |
| **Sex** | -2.68 | -4.0 to -1.40 | -0.53 | **< 0.001** |
| **BMI (kg/m^2^)** | -0.02 | -0.13 to 0.08 | -0.06 | 0.67 |
| **SBP (mmHg)** | -0.01 | -0.04 to 0.03 | -0.06 | 0.69 |
| **AVA (cm^2^)** | 0.61 | -3.05 to 4.28 | 0.05 | 0.74 |
| **Multivariate Analysis for WTd** | | | | |
| **Variable** | Unstandardized β Coefficient | 95% Confidence Interval | Standardized β Coefficient | p value |
| **Age (years)** | -0.01 | -0.07 to 0.06 | -0.02 | 0.89 |
| **Sex** | -2.80 | -4.21 to -1.39 | -0.55 | **< 0.001** |
| **BMI (kg/m^2^)** | 0.02 | -0.09 to 0.12 | 0.05 | 0.75 |
| **SBP (mmHg)** | 0.01 | -0.03 to 0.04 | 0.06 | 0.71 |
| **AVA (cm^2^)** | 0.07 | -3.55 to 3.68 | 0.04 | 0.97 |

Biological sex (male/female/intersex) ascertained by self-report.

Model performance/significance: R = 0.54, R^2^ = 0.29, F(5,42) = 3.37 with p = 0.01

Significant associations (p < 0.05) illustrated in bold.

WTd: wall thickness in end-diastole; BMI: body mass index; SBP: systolic blood pressure; AVA: aortic valve area.

**Supplementary Table 2**

**Suppl. Table 2. Simple linear regression analysis for the validation of non-invasive ESWS.**

| **Simple Linear Regression: Non-invasive ESWS Validation** | | | | |
| --- | --- | --- | --- | --- |
| **Variable** | Unstandardized β Coefficient | 95% Confidence Interval | Standardized β Coefficient | p value |
| **ESWS (x10^3^ dynes/cm^2^)** | 2.43 | 1.46 to 3.40 | 0.81 | **< 0.001** |
| **LVESP (mmHg)** | 0.33 | -0.02 to 0.67 | 0.46 | 0.06 |
| **Mean Gradient (mmHg)** | 0.86 | 0.64 to 1.08 | 0.90 | **< 0.001** |
| **SBP (mmHg)** | 0.70 | 0.16 to 1.23 | 0.58 | 0.01 |

Significant associations (p < 0.05) illustrated in bold.

ESWS: end-systolic wall stress; LVESP: left ventricular end-systolic pressure; SBP: systolic blood pressure.

**Supplementary Figure 1**


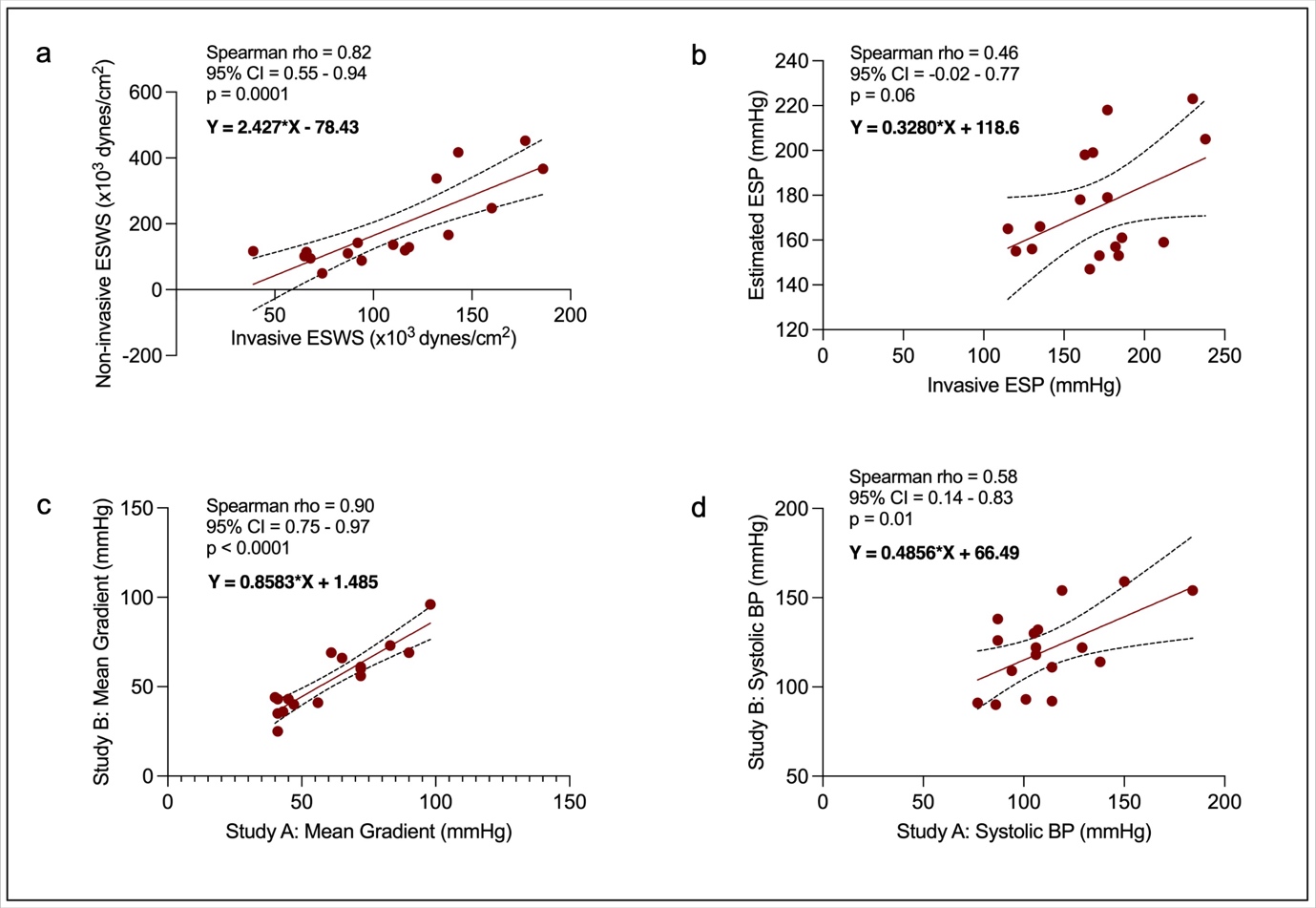


**Suppl. fig. 1.** Simple linear regression scatter plots demonstrating the strengths of linear association between (a) invasively measured ESWS and non-invasively derived ESWS, (b) invasively measured LV ESP and non-invasively derived LV ESP, (c) mean transaortic gradients derived by two blinded, independent echocardiographers, and (d) systolic (cuff) blood pressures measured during invasive catheterization versus at the time of CMR when non-invasive ESWS was estimated.

CI: confidence interval; ESWS: end-systolic wall stress; ESP: end-systolic pressure in the left ventricle; BP: blood pressure.
