# Supplementary material for: Morphological and functional evaluation of the left ventricle in severe aortic stenosis with afterload mismatch: a South African single-centre, cross-sectional cardiovascular MRI-based study
Source: BMJ Open. 2026 Jun 24;16(6):e118642. doi: 10.1136/bmjopen-2026-118642 (PMC13295984; doi:10.1136/bmjopen-2026-118642)
Supplement: Supplementary file 2 [file bmjopen-2026-118642.reviewer_comments.pdf]

PEER REVIEW HISTORY

BMJ Open publishes all reviews undertaken for accepted manuscripts. Reviewers are asked to complete a checklist review form and are provided with free text boxes to elaborate on their assessment. These free text comments are reproduced below.

ARTICLE DETAILS

Title (Provisional)

Morphological and functional evaluation of the left ventricle in severe aortic stenosis with afterload mismatch: A South African single-center, cross-sectional cardiovascular magnetic resonance imaging-based study

Authors

Rajah, Megan Rian; Doubell, Anton; Herbst, Philip

VERSION 1 - REVIEW

**Reviewer**	**1**
**Name**	**Dack, Kyle**
**Affiliation**	**University of Bristol, MRC Integrative Epidemiology Unit**
**Date**	**09-Apr-2026**
**COI**	**None**

Statistical review summary:

The paper adheres to STROBE and the methodology/results are reported well. The statistical analyses are implemented suitably but I do have two major concerns which I recommend making revisions regarding: (1) the abstract and methods reports this is a prospective study, but it is cross-sectional. (2) The comparisons were all bivariate correlations which did not adjust for other factors. Confounding bias due to sex, weight, age, etc is plausible, and the discussion would be strengthened if this was considered more. Generally the discussion interprets the findings in a manner close to causal inference, which the statistical methods are unable to support, and it would benefit from hedging the conclusions more.

Abstract:

1. The abstract is very clear and contains all important elements of the design, methods, and findings (no suggestions).

2. Strengths/limitations: One key risk which is not mentioned, or in the main text, is that confounding bias is a risk. Correlational analyses were used, which can not adjust for factors which influence both compared features. For example, age, sex, body size, possibly cormorbidities such as hypertension and CVD (this may be controlled for with the exclusion criteria), could systematically influence the compared factors, and the correlations could in part be artefacts from this.

Introduction:

3. No suggestions.

Methods:

4. Study design - it is reported to be a prospective study design in the abstract, and the main text reports that participants were prospectively followed. But it is not clear what the longitudinal element to the study is - what was the length of followup, and which variables were measured at later dates?

5. STROBE item 7: Variables - this is marked as NA, but there must be variables for there to be statistical comparisons. Pages 8-10 already describe variable measurements, integrated with descriptions of the data sources, and I recommend marking this as complete in the STROBE checklist.

6. Statistical analysis: The statistical approach is clearly described. However, it does not account for the risk of confounding bias. There are several factors - age, BMI, blood pressure, sex - which could influence both of the variables being compared. Without statistical adjustment, some of the correlations could be reflections of the shared influence of these factors, rather than reflecting a direct link between the variables themselves. Because of this I recommend more caution in the interpretation, and discussion of the degree to which the results could be reflective of confounding (currently it is not mentioned).

Results:

7. Table 1,2. For clarity I suggest adding as a footnote the statistical test the p-values were derived from.

8. Table 1. The percentage for males in the LEF-HG group appears incorrect - it is reported as 43% but looks to be around 33%.

Discussion:

9. The interpretation appears quite strong given that confounding factors were not adjusted for. There is also some causal interpretation, which could not be supported by the observational data. In multiple places, mechanisms are reported, which seems to go beyond what the data supports.

Additionally, it is reported that the study is cross-sectional - but earlier sections framed it as prospective. With a cross-sectional design, the direction of effect is unclear, and it is statistically plausible for the outcomes are influencing the exposures. While it is acknowledged in the limitations that causal inference cannot be made from this data, such inference is present throughout the discussion, which I recommend making more cautious.

**Reviewer**	**2**
**Name**	**Marlevi, David**
**Affiliation**	**Karolinska Institute**
**Date**	**12-Apr-2026**
**COI**	**None**

In this work, the authors used CMR to assess the relationship between LV remodelling and systolic function (defined together by cavity dimension, wall thickness, and wall stress) in an aortic stenosis cohort. Performed as a single-centre prospective assessment, and performed in a comparably small cohort, some concerns remain as per below:

• Afterload mismatch, as also noted by the authors, is a well described phenomenon. While predominantly assessed using other modalities, CMR studies do exist, not least from the submitting manuscript team itself. Novelty and the specific contribution of this work thus need to be better quantified, and mentioned explicitly in the Introduction.

• A value of CMR as compared to other modalities is its ability to quantify hemodynamics at higher accuracy than e.g. echo Doppler. Specifically, this stems from being able to use higher-dimensionality phase-contrast sequences (2D PC-MRI; or even 4D Flow MRI) to include jet eccentricity, intracavitary vortices, and so on. Was any phase-contrast data acquired? And if so, could the authors quantify the aortic stenosis status from the CMR as well? It has been well reported how echo-measurements deviate from invasive reference pressures *in particular* in stenotic valve configurations.

• The derivation of ESWS is critical, and requires further description. Reichek et al. performed assessment by M-mode diameter measures, in combination with cuff estimates to get a simplified stress measure. Here, the authors however include adjustment both moving into a CMR-setting (for which differences should be explained) as well as adding pressure variations accounted by the AS. This needs critical examination. Was the pressure gradient determined by CMR or echo? If the latter, again numerous work showcase the limitations of simplified Doppler-based (Bernoulli) assessments of pressure gradients, not least in an AS setting (see

e.g. Donati et al., Circulation: CVI, 2017). The authors should critically discuss this, but also perform evaluations on the robustness of their outcome. What if measures of pressure gradient vary by certain percentage? How does this affect results? And so on.

• Throughout the authors perform multiple testing for LEF-HG vs NEF-HG differences. P-value corrections should here be considered.

• Could the authors perform a multivariable regression assessment as to which parameters associate with ESWS? Not single-variable assessment are reported, but it would be highly relevant to assess multivariate relations, not least to see whether ESWS is an independent predictor of LEF-HG or whether it co-variates with other underlying markers

---

VERSION 1 - AUTHOR RESPONSE

Reviewer 1: Dr Kyle Dack

Statistical review:
The paper adheres to STROBE and the methodology/results are reported well. The statistical analyses are implemented suitably but I do have two major concerns which I recommend making revisions regarding (1) the abstract and methods reports this is a prospective study, but it is cross sectional. (2) The comparisons were all bivariate correlations which did not adjust for other factors. Confounding bias due to sex, weight, age, etc is plausible, and the discussion would be strengthened if this was considered more. Generally, the discussion interprets the findings in a manner close to causal inferences, which the statistical methods are unable to support, and it would benefit from hedging conclusions more.

Abstract:

1. The abstract is very clear and contains all important elements of the design, methods, and findings (no suggestions).

2. Strengths/limitations: One key risk which is not mentioned, or in the main text, is that confounding bias is a risk. Correlational analyses were used, which cannot adjust for factors which influence both compared features. For example, age, sex, body size, possibly comorbidities such as hypertension and CVD (this may be controlled for with the exclusion criteria), could systematically influence compared factors, and the correlations could in part be artefacts from this.
To strengthen the article and account for confounding bias, we have replaced the correlational analyses with simple linear and multivariate regression analyses (these are described in further detail under point number 6).

Introduction:

3. No suggestions.

Methods:

4. Study design – it is reported to be a prospective study design in the abstract, and the main text reports that participants were prospectively followed. But it is not clear what the longitudinal element to the study is – what was the length of followup, and which variables were measured at later dates?

   The study design was cross-sectional. We have corrected this in the abstract and main text (Abstract: page 2 and methods section: page 6).

5. STROBE item 7: Variables – this is marked as NA, but there must be variables for there to be statistical comparisons. Pages 8-10 already describe variable measurements, integrated with descriptions of the data sources, and I recommend marking this as complete in the STROBE checklist.

   Thank you for highlighting this. We have marked this item as complete in the updated STROBE checklist.

6. Statistical analysis: The statistical approach is clearly described. However, it does not account for the risk of confounding bias. There are several factors – age, body mass index, blood pressure, sex – which could influence both of the variables being compared. Without statistical adjustment, some of the correlations could be reflections of the shared influence of these factors, rather than reflecting a direct link between the variables themselves. Because of this I recommend more caution in the interpretation, and discussion of the degree to which the results could be reflective of confounding (currently it is not mentioned).

   To account for confounding bias, we have replaced the correlation analysis with simple linear and multivariate regression analysis Methodology described under the statistical analysis section on page 9). Given the centrality of wall thickness, left ventricular ejection fraction (LVEF), and wall stress (ESWS) to the research question posed, we have tested four multivariate models summarized as follows:

   - The associations between age, sex, body mass index, blood pressure, valve area and wall thickness were evaluated (Supplementary section: suppl. table 1). Sex was found to be significantly associated with wall thickness (a well-described association). This was discussed briefly in the discussion section (page 16).
   - The associations between age, sex, body mass index, blood pressure, valve area, ESWS and LVEF were evaluated to demonstrate that LVEF was independently related to ESWS (Results section: pages 13-14 and table 4).
   - The associations between age, sex, body mass index, systolic blood pressure, valve area, LV dimensions (cavity size and LV hypertrophy) and ESWS were evaluated as a direct test of our hypothesis (Results section: page 14-15 and table 5). Two separate models were evaluated – one that utilized two-dimensional measurements and another, that used three-dimensional measurements. These parameters could not be tested in a single model due to multicollinearity.

Results:

7. Table 1,2. For clarity, I suggest adding as a footnote the statistical test the p-values were derived from.
   We have included the p-value test derivations as a footnote in tables 1 and 2 (Results section: pages 10 and 12)

8. Table 1. The percentage for males in the LEF-HG group appears incorrect – it is reported as 43% but looks to be around 33%.
   Thank you for cross-checking these values. We have corrected this typo from 43% to 33% (Results section: page 10).

Discussion:

9. The interpretation appears quite strong given that confounding factors were not adjusted for. There is also some causal interpretation, which could not be supported by the observational data. In multiple places, mechanisms are reported, which seems to go beyond what the data supports. Additionally, it is reported that the study is cross-sectional – but earlier sections framed it as prospective. With a cross-sectional design, the direction of effect is unclear, and it is statistically plausible for the outcomes are influencing the exposures. While it is acknowledged in the limitations that causal inference cannot be made from this data, such inference is present through the discussion, which I recommend making more cautious.

   Thank you for this feedback. We have included adjusted analyses as described in point 6 above to strengthen the results, discussion and conclusion of the article. We have also amended the discussion and conclusion where relevant, being more cautious in our interpretation of parameters/variables whose temporal relationships are unknown by nature of the study design and have also emphasized this limitation more explicitly in the discussion (Discussion: pages 17-18).

Reviewer 2: Dr David Marlevi

Summary:

In this work, the authors used CMR to assess the relationship between LV remodelling and systolic function (defined together by cavity dimension, wall thickness, and wall stress) in an aortic stenosis cohort. Performed as a single-center prospective assessment, and performed in a comparably small cohort, some concerns remain as per below:

1. Afterload mismatch, as also noted by the authors, is a well described phenomenon. While predominantly assessed using other modalities, CMR studies do exist, not least from the submitting manuscript team itself. Novelty and the specific contribution of this work thus need to be better quantified, and mentioned explicitly in the introduction.
   The introduction section has been amended to report on previous findings of our work and to more explicitly describe the new contributions presented in this paper (Introduction section:

pages 4-5).

2. A value of CMR as compared to other modalities is its ability to quantify hemodynamics at higher accuracy than e.g. echo Doppler. Specifically, this stems from being able to use higher-dimensionality phase-contrast sequences (2D PC-MRI; or even 4D flow MRI) to include jet eccentricity, intracavitatory vortices, an so on. Was any phase-contrast data acquired? And if so, could the authors quantify the aortic stenosis status from CMR as well? It has been well reported how echo-measurements deviate from invasive reference pressures "in particular" in stenotic valve configurations.

   Thank you for sharing your insights. Two-dimensional phase contrast imaging at the aortic valve was performed on CMR. Our challenge with utilising the CMR images for an estimate of mean gradient is the presence of turbulence-associated aliasing that persists in spite of adjusted velocity encoding (adjusted up to 550 – 600 cm/s). Mitigation of turbulence was achievable during image acquisition by using a more distal imaging plane – however, this may not represent the true peak/mean gradient given its distance from the aortic valve tips. Unfortunately, we have no access to 4D flow.

   In a subset of this cohort, mean gradients were measured independently by two blinded, experienced echocardiographers using a Pedoff probe to ensure meticulous alignment with the AS jet. Using simple linear regression, we have confirmed a high correlation between the two groups of independent measurements – these results have been included in the supplement of the revised manuscript (Supplement section: suppl. table 2 and suppl. fig.1).

3. The derivation of ESWS is critical, and requires description. Reichek et al. performed assessment by M-mode diameter measures, in combination with cuff estimates to get a simplified stress measure. Here, the authors however include adjustment both moving into a CMR-setting (for which differences should be explained) as well as adding pressure variations accounted by the AS. This needs critical examination. Was the pressure gradient determined by CMR or echo? If the latter, again numerous work showcase the limitations of simplified Doppler-based (Bernoulli) assessments of pressure gradients, not least in and AS setting (see e.g. Donati et al., Circulation: CVI, 2017). The authors should critically discuss this, but also perform evaluations on the robustness of outcome. What if measures of pressure gradient vary by a certain percentage? How does this affect results? And so on.

   Thank you for this suggestion. Invasive cardiac catheterization is considered the gold standard technique for the assessment of wall stress. A subset of our cohort underwent cardiac catheterization with pressure-volume loop assessment for another study (Liebenberg et al., Heart, 2023). This data was invasively acquired utilising high fidelity micromanometer tipped pressure and conductance catheters (CD Leycom). We have correlated our non-invasive MRI derived measurements with the invasive measurements by Liebenberg et al., and found a strong linear relationship (rho = 0.81, 95% confidence interval 0.55 to 0.94, $p < 0.001$) between the measurements. We've also correlated invasive end-systolic pressure to our non-invasively estimated LV pressure that was derived by the sum of systolic cuff pressure and mean gradient from echo. The strength of association here was insignificant (rho = -0.46 with p = 0.06, noting

that the sample size was small) and this appeared to be driven by variations in systolic blood pressure rather than mean gradients. The range of differences in end-systolic pressure was 2 to 53 mmHg with a balance in terms of which method yielded higher pressures (8 with higher invasive pressures and 9 with higher surrogate measures). These results have been included in the supplement (Supplementary section: suppl. table 2 and suppl. fig. 1) and a statement regarding the correlation between invasive and non-invasive ESWS was included in the results section (page 13).

4. Throughout the authors perform multiple testing for LEF-HG vs NEF-HG differences. P-value corrections should here be considered.
   Thank you for this suggestion. Although the CMR variables tested and reported were specifically selected for the study's research question, we acknowledge the statistical limitation that may still exist with multiple testing. Therefore, we have re-analyzed our CMR variables using a Welch's ANOVA (this test was selected in view of heterogeneity in the variances of our data). Corrected p values are now reported in the revised manuscript and were calculated/derived using the Dunnett's T3 test. (Results: Table 2, pages 11-12). The statistical analysis section under the methodology has also been amended to reflect this change (page 9).

5. Could the authors perform a multivariable regression assessment as to which parameters associate with ESWS? Not single-variable assessment are reported, but it would be highly relevant to assess multivariate relations, not least to see whether ESWS is an independent predictor of LEF-HG or whether it co-variates with other underlying markers.
   Thank you for this suggestion. We have included a multivariate analysis to assess whether age, sex, systolic blood pressure, aortic valve area, and LV chamber dimensions were independently associated with ESWS. To ensure that the assumption of multicollinearity was met, two separate models were tested - one using the two-dimensional measurements (LV internal diameter and wall thickness) as reflected in Laplace's Law and the other, using three-dimensional volumetric measurements (indexed end-diastolic LV volume and LV mass). Both models are reported on in the results section (pages 14-15 and in table 5). The discussion has been amended where necessary to reflect these changes.

Thank you for the guidance on strengthening this manuscript.

VERSION 2 - REVIEW

**Reviewer**	**1**
**Name**	**Dack, Kyle**
**Affiliation**	**University of Bristol, MRC Integrative Epidemiology Unit**

**Date**	**02-Jun-2026**
**COI**	

The revisions resolved all of my suggestions or queries. The addition of multivariable regression is a strength and allows for clearer interpretation. However, the rationale for the regression was not stated in the paper itself, and readers may be unaware of what it adds. Confounding bias is also not mentioned directly in the paper, and in its absence, some readers may still interpret that it was not taken into consideration.

This could be clarified with a brief addition to the statistical analysis to explain the reason for the multivariable regression, the covariates adjusted for, and how they were selected (e.g. based on prior theory or evidence). Please also clarify the reference category for sex in the models.

**Reviewer**	**2**
**Name**	**Marlevi, David**
**Affiliation**	**Karolinska Institute**
**Date**	**29-May-2026**
**COI**	

The authors have answered all my questions in a satisfactory manner.

VERSION 2 - AUTHOR RESPONSE

Reviewer 1: Dr Kyle Dack

1. The revisions resolved all of my suggestions or queries. The addition of multivariable regression is a strength and allows for clearer interpretation. However, the rationale for the regression was not stated in the paper itself, and readers may be unaware of what it adds. Confounding bias is also not mentioned directly in the paper, and in its absence, some readers may still interpret that it was not taken into consideration. This could be clarified with a brief addition to the statistical analysis to explain the reason for the multivariable regression, the covariates adjusted for, and how they were selected (e.g. based on prior theory or evidence).
Thank you for this additional suggestion. We have included further details in the statistical analysis section of the methods (page 9) to explain why a multivariate analysis was performed. We have also included a list of the confounding variables selected for inclusion in the multivariate models.

2. Please also clarify the reference category for sex in the models.
Biological sex by self-report was used to categorize sex for the study. We have included this as a footnote in table 1 (page 10), table 4 (page 14), table 5 (page 15) and supplementary table 1.

Reviewer 2: Dr David Marlevi

The authors have answered all my questions in a satisfactory manner.

Thank you for reviewing our revisions and for your valuable insights during the peer review process.

Thank you for the guidance and academically enjoyable peer review process.
